# Supplementary material for: First field evaluation of novel LDH- and HRP2-based rapid tests for Plasmodium vivax and Plasmodium falciparum malaria diagnosis
Source: PLoS Negl Trop Dis. 2025 Oct 27;19(10):e0013307. doi: 10.1371/journal.pntd.0013307 (PMC12578339; doi:10.1371/journal.pntd.0013307)
Supplement: S1 File — (PDF) [file pntd.0013307.s001.pdf]

First Field Evaluation of Novel LDH- and HRP2-based Rapid Tests for *Plasmodium vivax* and *Plasmodium falciparum* Malaria Diagnosis

Supplementary File S1: qPCR and dPCR protocols

P. falciparum varATS qPCR

Reference: Ultra-sensitive detection of Plasmodium falciparum by amplification of multi-copy subtelomeric targets.
Hofmann N, Mwingira F, Shekalaghe S, Robinson LJ, Mueller I, Felger I
PLoS Medicine 2015

Prepare primers and probe to **10 uM**

varATS forward+reverse	0.48 uL	
varATS_probe	0.48 uL	
VWR PerfeCTa Though Mix	6 uL	Alternative: ThermoFisher FastAdvanced MM
H2O	1.04 uL	
DNA	4 uL	
Total	12 uL	

varATS forward	cccatacacaaccaaytgga
varATS reverse	ttcgcacatatctctatgtctatct
varATS probe	6-FAM-trttccataaatggt-NFQ-MGB

This is a minor groove binding probe. Order from Thermo Fisher Scientific (RNAi, Oligos & Assay Tools > Applied Biosystems Customs TaqMan Probes)

50° 2 min
95° 2 min
95° 10 sec
55° 30 sec => 45 cycles

P. vivax cox1 qPCR

Reference: Plasmodium vivax molecular diagnostics in community surveys: pitfalls and solutions.
Gruenberg M, Moniz CA, Hofmann NE, Wampfler R, Koepfli C, Mueller I, Monteiro WM, Lacerda M, de Melo GC, Kuehn A, Siqueira AM, Felger I
Malaria Journal 2018

Prepare primers and probe to **10 uM**

Cox1 forward+ reverse	0.48 uL
Cox1 probe	0.48 uL
FastAdvanced MM	6 uL
H2O	1.04 uL
DNA	4 uL
Total	12 uL

50° 2 min
95° 2 min
95° 10 sec
60° 30 sec => 45 cycles

Pv_Cox1 forward	5’-TTATATCCACCATTAAGTACATCACTT-3’
Pv_Cox1 reverse	5’-AACCTTTAGATCTTAGATGCATTACA-3’
Pv_Cox1 probe	5'-VIC-CCTGTTGCAGTAGATGTTATCATTG-BHQ1-3’

hrp2 exon 2 / *hrp3* / *tRNA* QIAcuity Protocol

Reference: High-throughput Plasmodium falciparum hrp2 and hrp3 gene deletion typing by digital PCR to monitor malaria rapid diagnostic test efficacy.
Claudia A Vera-Arias, et al
Elife. 2022;11:1-16. doi:10.7554/eLife.72083

Note**:** This protocol was optimized from the reference above to allow multiplexing of *hrp2*, *hrp3* and *tRNA* in a triplex reaction on the QIAcuity digital PCR instrument.

Reaction set up

Working stock of All Primers are at **100 µM** concentration.
Working stock of All Probes are at **10 µM** concentration.

	1x [uL]
QIAcuity Probe PCR Kit	3
hrp2_ fwd (1.6 µM)	0.192
hrp2_ rev (1.6 µM)	0.192
hrp2_ Probe (0.1 µM)	0.12
hrp3_fwd (0.8 µM)	0.096
hrp3_rev (0.8 µM)	0.096
hrp3_Probe (0.05 µM)	0.06
tRNA_fwd (1.6 µM)	0.192
tRNA_rev (1.6 µM)	0.192
tRNA_Probe (0.1 µM)	0.12
H2O	5.75
DNA	2
Total	12

Cycling conditions (see below for screenshots of detailed input parameters):

95° 2 min
95° 15 sec
56° 1 min => 50 x back to step 2

hrp2: FAM
hrp3: TEXAS RED (ROX should be selected in QIAcuity machine)
tRNA: HEX

Primers + Probes

hrp2_fwd	5’-CATTTTTAAATGCTTTTTTATTTTTATATAG-3’
hrp2_rev	5’-CTTGAGTTTCGTGTAATAATCTC-3’
hrp2_probe	6-FAM/CGCATTTAATAATAACTTGTGTAGCAAAAATGC-3’
tRNA_probe	5’-HEX/CTACCTCAGAACAACCATTATGTGCT[BHQ1]-3’
tRNA_fwd	5’-CATCAAATGAAGATTTAACAAGAG-3’

tRNA_rew 5’-CTTTTTGATTCTATAGTTTCATCTTTATG-3’

hrp3_fwd 5’-ATGCTAATCACGGATTTCATTTTA-3’
hrp3_probe 5’-TEX/CCTTCACGATAACAATTCCCATACTTTAC-3’
hrp3_rev 5’-ATCGTCATGGTGAGAATCATC-3’

Input cycling protocol for the QIAcuity One

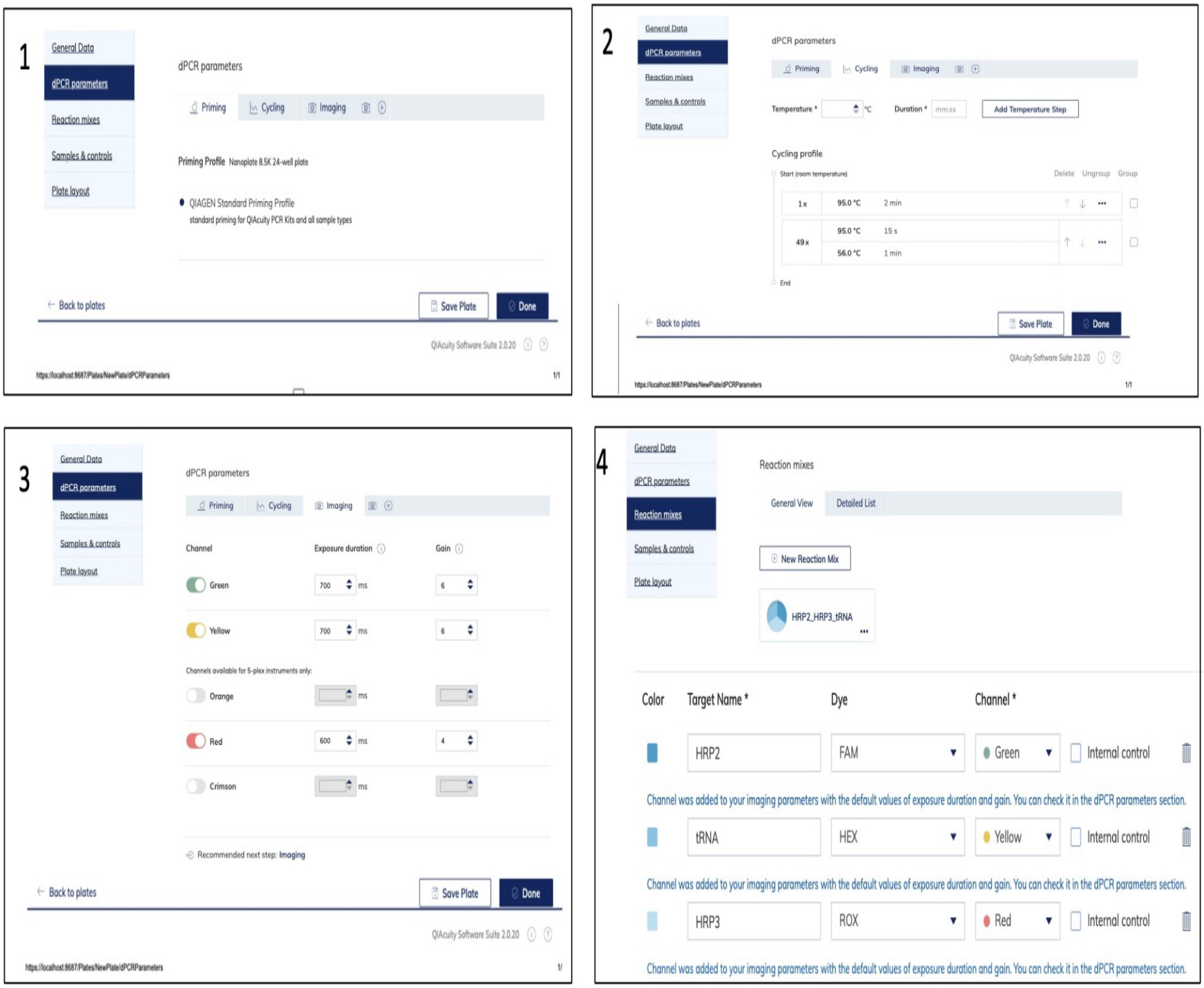

Examples of Results

1-D separation

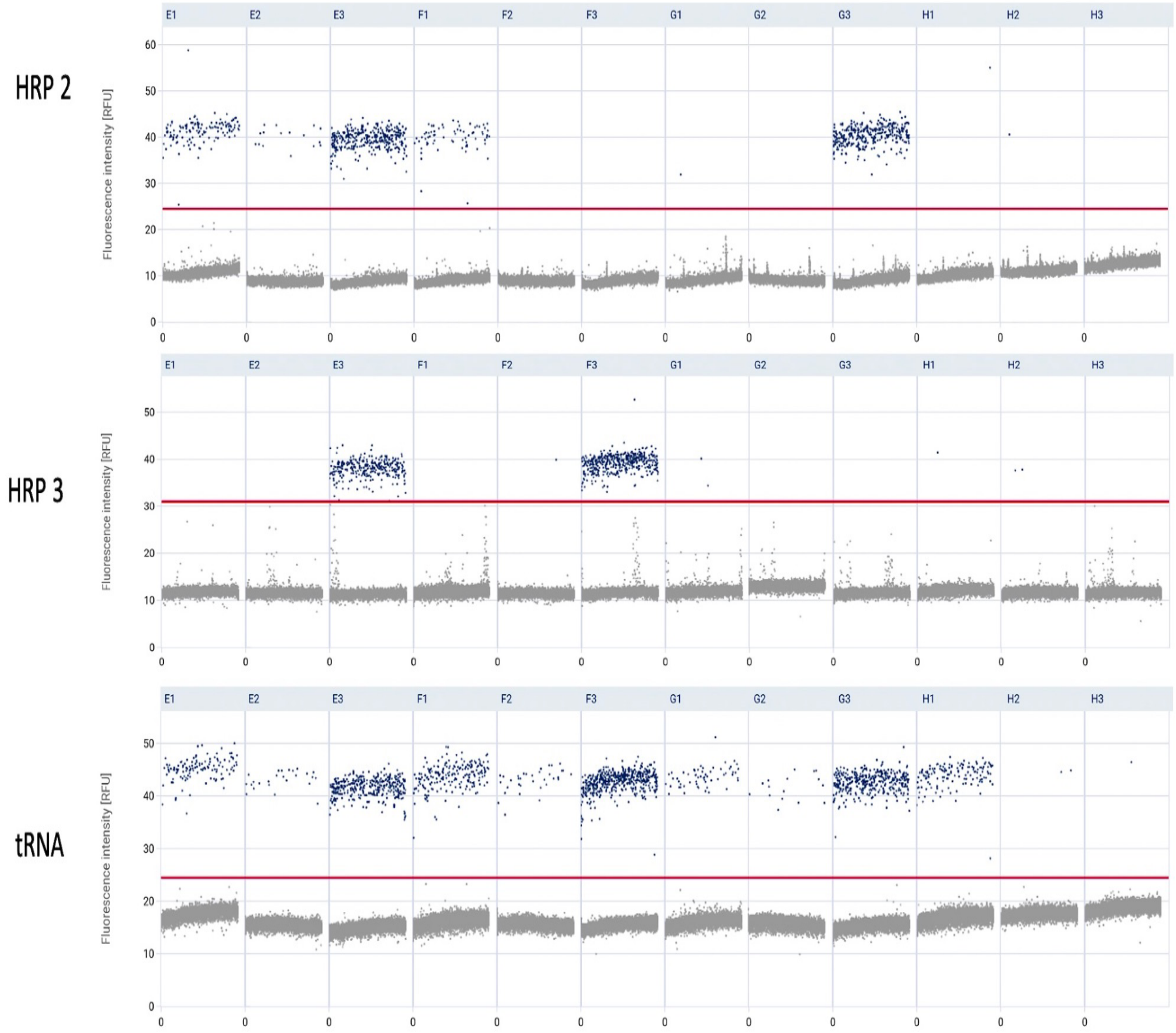

2-D separation

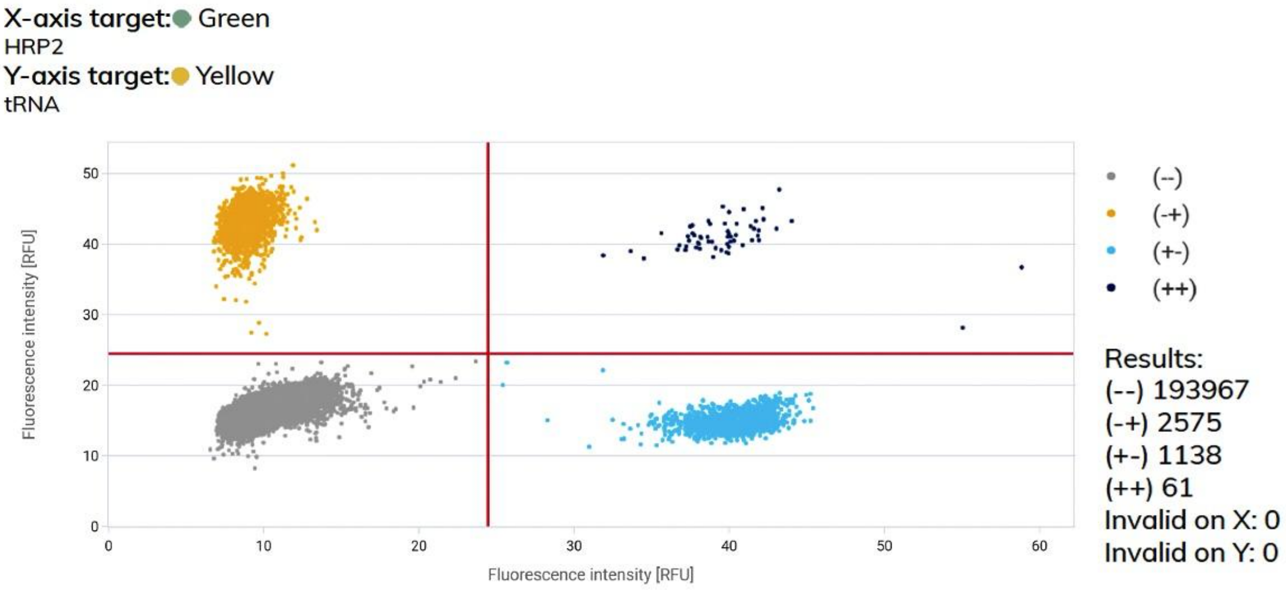

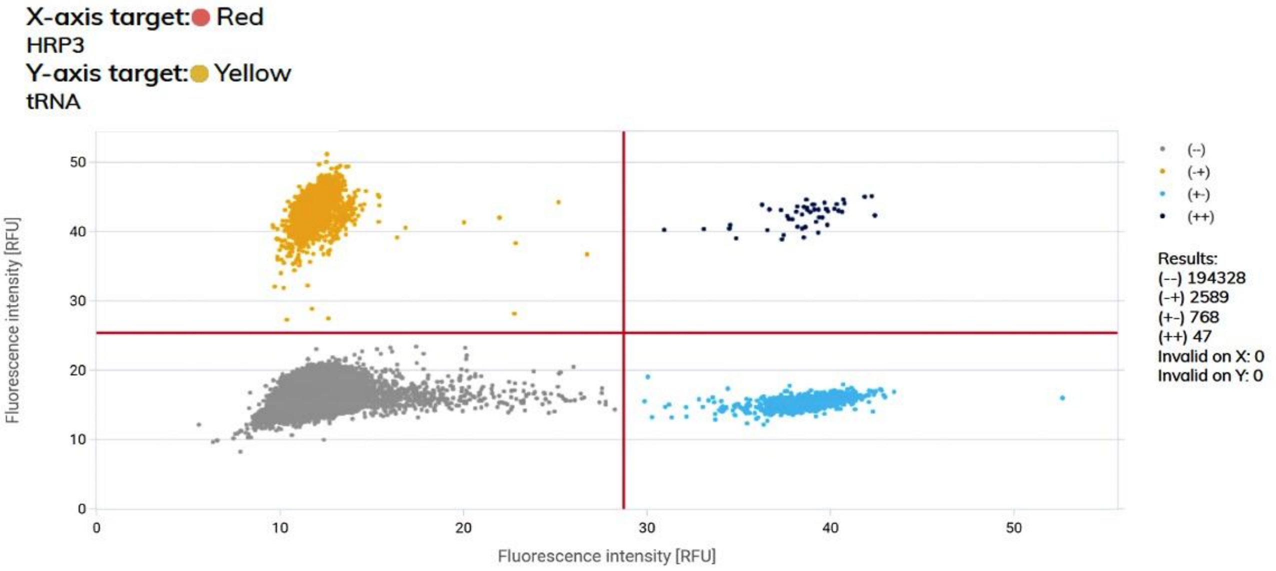

Data analysis parameters

Include sample in analysis:	≥8 droplet positive for tRNA
Sample is *hrp2* negative	If density is ≥200 droplets positive for tRNA: <1% hrp2-positive droplets compared to tRNA (i.e., a minimum of 2 droplets positive for *hrp2*). This threshold is set to account for possible cross-contamination. The 1% threshold might be adjusted for very high-density samples.<br>If density is <200 droplets positive for tRNA: 0 or 1 droplets positive for *hrp2*

Sample contains hrp2	If density ≥100 droplets positive for tRNA: ≥1% droplets positive for hrp2 compared to tRNA<br>If density <100 droplets positive for tRNA: ≥2 droplets positive for hrp2
Mixed infection:	≥50 droplets positive & <60% hrp2 compared to tRNA<br>Mixed infections cannot be reliably detected at low densities.
