# Supplementary material for: First field evaluation of novel LDH- and HRP2-based rapid tests for Plasmodium vivax and Plasmodium falciparum malaria diagnosis
Source: PLoS Negl Trop Dis. 2025 Oct 27;19(10):e0013307. doi: 10.1371/journal.pntd.0013307 (PMC12578339; doi:10.1371/journal.pntd.0013307)
Supplement: Attachment — Submitted filename: Response to reviewers.docx [file pntd.0013307.s005.docx]

We thank all reviewers for their detailed comments. Reviewer comments are in black. Our responses are in blue.

Our preprint was selected by RR/ID for a review. Two reviews can be found here: <https://rrid.mitpress.mit.edu/pub/6qy4d9xs/release/1>. We also addressed some comments raised there. In particular, we clarified

- That patients with signs of severe malaria were excluded (line 103)
- How long blood had been kept at -20°C before testing with the Bioline RDT (line 141)
- That expert microscopists were blinded to qPCR results (line 151).

Reviewer 1

Overall, this is a good study and I have only a minor suggestion:

The current title of the manuscript requires revision. It can be modified along the lines of “First Field Evaluation of BIOCREDIT Malaria Ag Pf (cHL) and BIOCREDIT Malaria Ag Pf/Pf/Pv (cHL) Rapid Tests for Plasmodium vivax and Plasmodium falciparum Malaria Diagnosis”

Response: Going through the literature on RDT testing, it seems unusual to include the RDT brand names in the title. Further, we do not believe the readers would easily grasp that these RDTs detect HRP2 and LDH. We thus prefer to leave the title as is.

Reviewer 2

The authors present the results of a prospective study comparing different RDTs for *P. falciparum* and/or *P. vivax* among 708 clinical patients enrolled at three health centers in southern Ethiopia. This study addresses the problem of diagnosis of malaria in patients infected with parasites that have hrp2/3 gene deletions, compares the diagnosis of *P. falciparum* and *P. vivax* infections, and is the first field test of the BIOCREDIT Ag Pf (cHL) and BIOCREDIT Ag Pf/Pv (cHL/L) RDTs.

Specific Comments:

1. In the Introduction, the authors need to highlight what exactly makes their study unique and novel and stand out compared to other RDT studies.

Response: We added the following detail (lines 87-89):

“The of the RDTs (BIOCREDIT Malaria Ag Pf (cHL) and BIOCREDIT Malaria Ag Pf/Pv (cHL/L)) had not previously been tested in the field. Few studies directly compared RDTs with separate HRP2 and LDH lines to those with combined lines [12].”

2. Referring to lines 31-32 and 233-235, please clarify whether typing of hrp2/3 deletions was conducted on 53 *P. falciparum* infected samples or on all *Plasmodium* infected samples.

Response: We clarified the abstract as follows:

“Only 2/52 *P. falciparum* infections typed carried *hrp2* and *hrp3* deletions, both were detected by all LDH-based RDTs.

3. Please consider deleting “consecutive” from line 82.

Response: This term is required by STARD guidelines. While we agree it might be obvious from our description of sample collection, we prefer to leave at is.

4. By qPCR, 10.9% carried *P. falciparum* mono-infections, 28% carried *P. vivax* mono-infections, and 7.2% carried mixed *P. falciparum* and *P. vivax* infections (lines 192-194). By microscopy, 5.8% were *P. falciparum* mono-infections, 13.6% were *P. vivax* mono-infections, and 0.3% were mixed infection. Please explain the more precipitous drop in sensitivity to detect mixed infections between the two methods compared to mono-infections.

Response: Low sensitivity of microscopy to detect both species in mixed-infections is a well-known phenomenon. We added the following to the results and discussion:

Results (lines 295-298):

“Among 12 samples with *P. falciparum* and *P. vivax* mixed-infection, each at >20 parasites/µL, both species were detected in only one sample by health center microscopy. In contrast, the BIOCREDIT RDT detected 5/12, and the Bioline RDT detected 7/12 mixed-species infections. By expert microscopy, five mixed-infections were detected.”

Discussion (lines 316-320):

“As seen in other studies [22, 23], sensitivity of health center microscopy was particularly low for mixed-species infections. RDTs performed better and detected both species in 5/12 (BIOCREDIT) and 7/12 (Bioline) infections where *P. falciparum* and *P. vivax* were present at densities >20 parasites/µL. While this is well below the >90% sensitivity for either species across the entire dataset, the number of samples is too low to determine RDT performance for mixed-species infections.“

5. Provide an explanation for why the LOD for the LDH-based tests is substantially higher for *P. vivax* compared to *P. falciparum* (lines 270-273).

Response: We believe the reviewer misread the text. The LOD for Pf LDH and Pv LDH is very similar at approx. 200 parasites/µL. The text states:

“The limit of detection of HRP2-based tests for *P. falciparum* was in the range of 30-60 parasites/µL, compared to approximately 250 parasites/µL for the LDH line on the Bioline RDT. The limit of detection of LDH-based tests for *P. vivax* was substantially higher, i.e., approximately 200 parasites/µL on the Bioline and BIOCREDIT RDTs.”

6. In line 281, replace “an” with “a”.

Response: Done

7. In lines 293-295, “sensitivity” is not the appropriate word to use.

Response: We are not sure why the reviewer thinks that this word is not appropriate. We clarified that we mean sensitivity of RTD vs. qPCR (lines 339-340):

“Sensitivity vs. qPCR for both lines combined was 52% in Ghana [13], and thus similar to the combined line of the test evaluated in the current study. In Burundi sensitivity vs. qPCR was 80% [12].”

Reviewer 3

1. Insufficient methodology has been provided for the qPCR and digital PCR assays. While references are available, it would be much clearer if this manuscript included primer sequences, reaction conditions, volume, equipment and reagents used.

Response: We added Supplementary File S1 that contains all protocols.

We added on line 178: “All protocols for qPCR and dPCR are provided in Supplementary File S1.”

The other supplementary files were renamed Supplementary File S2 and Supplementary File S2.

2. Is the impact of freezing blood on the sensitivity of the Bioline RDT known? This should be discussed as a possible impact.

Response: We added the following to the methods (line 141-143):

“While freezing is not recommended by WHO guidelines for RDT testing and might impact sensitivity, a single freeze-thaw cycle as done for this study was shown not to affect HRP2 concentrations [15].”

3. Line 216. It is not clear how the limit of detection was determined.

Response: We added the following detail to the methods section (lines 202-209):

The limit of detection (LoD) was defined as the lowest parasite density at which the probability of a positive RDT result reached 95%. The binary RDT outcome (positive/negative) was modeled as a function of log10-transformed parasite densities by qPCR, using a generalized linear model with a probit link, which models detection probability as the cumulative normal distribution of parasite density. The fitted model provided a detection probability curve, and the LoD was identified as the parasite density at which this curve crossed the 95% detection probability. Confidence intervals for the LoD were calculated by numerically solving for the parasite densities where the 95% confidence bounds of the fitted curve intersected the 95% detection probability.

4. It would be helpful to add a column in the tables that includes only samples with less than 20 parasites/ul, since it is difficult to determine without looking at supplementary files and will make it clearer to the reader that the sensitivity is very low at these densities. Alternatively, this could be described (using quantitative data) in the discussion when mentioning differences between studies and the LOD of qPCR methods used.

Response: We are a bit puzzled by this comment. As the reviewer points out, RDTs are not expected to detect such very-low density samples, and calculations of sensitivity at such low density is primary influenced by the limit of detection of the PCR. Rather than adding a column to the table, we added the following sentence to the results (line 294):

“For either species, at densities <20 parasites/µL, as expected, RDTs showed poor sensitivity of below 10%.”

5. Line 173, it mentions 7 samples that were microscopy positive but PCR negative. Have species other than P. falciparum and P. vivax been considered and how they would impact RDT results? Is it known if they cross-react?

Response: We agree this is a possibility, but did not screen by qPCR for other species. Currently, assays for *P. malariae* and *P. ovale* are not established in our PCR lab in Hawassa, thus screening for these species is not feasible for this study.
