# Supplementary material for: First field evaluation of novel LDH- and HRP2-based rapid tests for Plasmodium vivax and Plasmodium falciparum malaria diagnosis
Source: PLoS Negl Trop Dis. 2025 Oct 27;19(10):e0013307. doi: 10.1371/journal.pntd.0013307 (PMC12578339; doi:10.1371/journal.pntd.0013307)
Supplement: Attachment — Submitted filename: Response_to_reviewers_auresp_2.docx [file pntd.0013307.s006.docx]

Reviewer/editor comments are in black. Our responses are in blue.

Reviewer 3

It would be helpful to add a column in the tables that includes only samples with less than 20
parasites/ul, since it is difficult to determine without looking at supplementary files and will make
it clearer to the reader that the sensitivity is very low at these densities. Alternatively, this could
be described (using quantitative data) in the discussion when mentioning differences between
studies and the LOD of qPCR methods used.

Author Response: We are a bit puzzled by this comment. As the reviewer points out, RDTs are not
expected to detect such very-low density samples, and calculations of sensitivity at such low
density is primary influenced by the limit of detection of the PCR. Rather than adding a column to
the table, we added the following sentence to the results (line 294):

“For either species, at densities <20 parasites/µL, as expected, RDTs showed poor sensitivity of
below 10%.”

Editor comments. Please add a column in the table as suggested by the reviewer. Your response to this comment is not acceptable.

Adding this data to existing tables would be misleading. First, none of the tables contains data with an upper limit in density, such as 0-20 parasites/µL, as you request. Rather, they include data for samples at densities >0, >20, and >200 parasites/µL. Adding such data could easily be misread by the reader. Second, we do not see any scientific justification for giving these data such prominence. It is very well known that ultrasensitive qPCR, as we conducted, can detect many more infections than RDTs. No RDT is meant to detect very low-density infections. What is the rationale to include these data? Shouldn’t data be presented based on a scientific question? Please note that even the reviewer proposed to include the data either in a table or in the text.

To address this comment, we expanded our text as follows (lines 298-302):

“At densities <20 parasites/µL, as expected, RDTs showed poor sensitivity. For *P. falciparum*, the BIOCREDIT Malaria Ag Pf (cHL) detected 4.4% (3/68), the BIOCREDIT Malaria Ag Pf/Pv (cHL/L) 5.9% (4/68), the Bioline Malaria Ag Pf/Pf/Pv 2.9% (2/68), and the First Response 5.3% (2/38) of infections. For *P. vivax*, the BIOCREDIT Malaria Ag Pf/Pv (cHL/L) detected 1.1% (1/128), the Bioline Malaria Ag Pf/Pf/Pv 1.6% (2/128), and the First Response 1.1% (1/94) of infections.“

5. Line 173, it mentions 7 samples that were microscopy positive but PCR negative. Have species
other than P. falciparum and P. vivax been considered and how they would impact RDT results? Is
it known if they cross-react?

Response: We agree this is a possibility, but did not screen by qPCR for other species. Currently,
assays for P. malariae and P. ovale are not established in our PCR lab in Hawassa, thus screening
for these species is not feasible for this study.

Editor Comment: Please include a line in the results that no PCR testing was not done for P. malariae and P. ovale in this study.

Response: This was added on line 164: “No qPCR screening for *P. malariae* or *P. ovale* was done.”
